# A Rasch Analysis of Students’ Academic Motivation toward Mathematics in an Adaptive Learning System

**DOI:** 10.3390/bs12070244

**Published:** 2022-07-21

**Authors:** Lyndon Lim, Seo Hong Lim, Wei Ying Rebekah Lim

**Affiliations:** Teaching & Learning Centre, Singapore University of Social Sciences, Singapore 599494, Singapore; shlim@suss.edu.sg (S.H.L.); rebekahlimwy@suss.edu.sg (W.Y.R.L.)

**Keywords:** academic motivation, validation, mathematics education, Rasch

## Abstract

Despite the criticality of considering student academic motivation as it influences learning, research within the field of adaptive learning technologies for education has so far focused more on customising instruction to implement personalised learning, than investigating how personalised learning is associated with learners’ motivation. Given this, a robust instrument is required to gather information about student academic motivation within the context of adaptive learning technologies. This study sought to validate the Academic Motivation Toward Mathematics Survey (AMTMS) currently used to measure motivation based on self-determination theory in mathematics education at pre-tertiary levels (grades 11 and 12) in Asia. A total of 196 participants recruited via availability sampling took part in this study, after interacting with an in-house mathematics adaptive learning system within a tertiary educational institution. The validation was performed based on modern test theory given that it overcomes some limitations of classical factor analytic approaches. Results supported the factorial structure of the AMTMS but 12 of the original 21 items had to be rescored to establish ordered thresholds. Further, the bifactor equivalent solution suggested the possibility of reporting a singular motivation index comprising the five factors within the AMTMS. Along with the results, this study offers researchers a robust and validated instrument to measure motivation toward mathematics that can be used within an adaptive learning environment.

## 1. Introduction

To improve students’ learning experiences and outcomes, tertiary educational institutions are increasingly adhering to technologies such as adaptive learning systems. Adaptive learning coursewares use computer algorithms to parse data gathered during students’ interaction with e-learning environments to adapt learning modules, instruction, and assessment [1].

Studies on adaptive technology have found mixed results in terms of academic performance, though this field of research is relatively nascent [1,2]. Some have also indicated that academic motivation has been shown to have an effect on the impact of adaptive learning technologies [3,4]. Despite the criticality of student motivation in learning analytics research, research has so far focused more on customising instruction to implement personalised learning, than investigating how personalised learning is associated with learners’ intrinsic motivation, competence, and autonomy [5,6,7,8,9].

With the growing trend of e-learning, including the use of adaptive learning systems in various higher learning institutions, it is worthwhile to gain insights on student motivation as they interact with adaptive learning systems [10,11]. To the authors’ knowledge and based on the literature review undertaken as part of this study, there remains a dearth of published instruments measuring tertiary students’ academic motivation validated using Rasch measurement theory (RMT) within the context of an adaptive learning system. To fill this gap, the current study aimed to validate the Academic Motivation Toward Mathematics Scale (AMTMS) [12] using RMT, with the view that a robust instrument would provide valuable information related to tertiary-student motivation as they interact with an in-house adaptive learning system (a.k.a. AdLeS). The AMTMS was selected as: (1) it had been validated with 1610 pre-tertiary (grades 11 and 12) students in Singapore, a country within which this study was conducted; (2) it presented psychometrically sound properties based on a sufficiently large sample, though these properties were established based on the classical factor analytic approach; (3) it comprised items appropriate and relevant to AdLeS; and (4) it is an abbreviated version of the AMS (i.e., 21 versus 28 items) and comprises five sub-scales (i.e., amotivation (AMOT), external regulation (EMER), introjection (EMIN), identification (EMID) and intrinsic motivation (IMT)) along the well-established self-determination continuum [13].

## 2. Motivation

Motivation is an important construct for researchers and educators due to its relation to learning and teaching contexts. A number of complex and multidimensional definitions have been proposed to explain motivation, though none have departed from how motivation was initially derived (i.e., from the Latin verb “movere” which means to move [14]). For example, [15] described motivation as the forces that drive and direct one’s behaviour towards a desired outcome; [16] defined motivation as that to be moved to take an action; while [17] stated that motivation can influence what, how, and when learners decide to learn.

According to established studies, motivated learners are more likely to actively engage in activities, and exhibit persistence in learning [16]. To enhance students’ motivation and provide insights for understanding learners’ actions, a vast amount of research continues to examine its role when assisting learners to succeed in various educational contexts [18]. Particularly for learning within online environments, motivation is vital [19,20], because unmotivated students tend to feel hopeless, and easily discontinue their learning processes [21]; this cements the importance of considering student motivation as a factor for success in online learning contexts [22,23]. Hence, the need to measure motivation via a robust instrument when learning takes place within an adaptive learning environment is non-trivial.

### 2.1. Self-Determination Theory (SDT)

Undergirding the study of motivation is the established self-determination theory (SDT), which proffers the notion that individuals have three fundamental psychological needs: autonomy, competence, and relatedness, and these either move individuals to act or not to act [24]. When these three needs are satisfied and supported, they can contribute to students’ motivation [25]. Subsequently, positive outcomes such as optimal motivation, internalisation, and learning will follow [13,26,27].

Ryan and Deci (2000) [16] further noted that these three needs experience varying degrees of motivational orientation, moving from amotivation, to extrinsic, and intrinsic motivations (see Table 1, which presents a synthesis of these motivational orientations). These needs also reflect students’ natural propensities to learn, since motivation differs in quality depending on relative autonomy, contributing to learners having different reasons for engaging in a behaviour [28].

### 2.2. Measuring Motivation in Learning Mathematics

There is a wealth of research on motivation-related constructs and their associations with mathematics instruction, achievement and educational outcomes. Instruments, some with unsatisfactory psychometric properties based on Messick’s seminal unified concept of validity, and their derivatives have been developed within certain contexts to assess various related constructs [29]. Nonetheless, the 28-item Academic Motivation Scale (AMS) by [30] is considered one of the most comprehensive and widely used instruments in assessing motivation from a self-determination-theory perspective [12]. The AMS has been tailored to fit various contexts (languages and grade levels) and disciplines. For example, it has been adapted into a Turkish version [31]; it has been validated against first year university mathematics students in Bulgaria [32], and against students from grades 9 to 12 offering biology [33].

While there are other motivation scales developed specifically for mathematics such as the Mathematics Motivation Scale by [34], and the Motivation for Mathematics Abbreviated Instrument by [29], these scales have been developed for a non-Asian and non-Singaporean context, and are not as established as the AMS. Given these reasons, this study adapted the AMTMS for use with AdLeS, and validated it using RMT.

### 2.3. Rasch Measurement Theory (RMT)

RMT is a unidimensional measurement model with a set of requirements (e.g., response dependency, dimensionality, etc.). Instead of focussing on explaining variance like the classical factor analytic approach, RMT provides a basis for measurement; observed data in Rasch analysis that do not fit the model would need to be adjusted to fit the model. The polytomous Rasch model is used to analyse data with more than two response categories and is expressed by Equation (1) [35].

(1) $$P\left\{x_{ni}=x\right\}=\frac{e^{-\tau_{1i}-\tau_{2i}\ldots -\tau_{\chi i}+\chi \left(\beta_{n}-\delta_{i}\right)}}{\sum_{\chi^{\prime }=0}^{m_{i}}e^{-\tau_{1i}-\tau_{2i}\ldots -\tau_{\chi^{\prime }i}+\chi^{\prime }\left(\beta_{n}-\delta_{i}\right)}}\;\;\;\;\;\;\;\;\;\;\;\;\;\;\;\;\;$$

where $P\left\{x_{ni}=x\right\}$ is the probability that person *n* selects in category “*x*”, respectively, on item *i*; *β* is the person location parameter; *τ* are the response probability thresholds; and *δ* is the mean of these thresholds. Based on Equation (1), RMT provides a table of expected response probabilities that reflect the theory (i.e., a person with greater proficiency should have a higher probability of solving or endorsing an item).

The AMTMS had been previously validated upon the classical factor analytic approach [12]. Nonetheless, RMT enables an examination of the hierarchical structure, unidimensionality, and additivity of the scores generated through rating scales by providing a foundation for doing so [36]. In RMT, item parameters are independent of respondent characteristics, and person parameters do not depend on the specific items within a questionnaire; validating an instrument upon RMT hence overcomes a limitation of the classical factor analytic approach, where measurements are not person- and item-distribution free [36,37]. Further, RMT offers the advantages of focusing on probabilistic distributions of respondents’ performance at the item- rather than test-level data by first locating and ordering individuals and item difficulty on a log-linear scale reflecting degrees of the latent trait (e.g., most to least endorsable).

## 3. Method

This study was conducted in two stages, both of which sought validity evidence for the AMTMS based on the unified concept of validity recommended by the Standards for Educational and Psychological Testing [38].

### 3.1. Stage 1

In Stage 1, the original 21-item AMTMS was reviewed for content appropriateness by two faculty leading and one professional staff member involved in mathematics education and AdLeS within the university. While the AMTMS was deemed as the most appropriate instrument for this study given the context under which it was developed and the semblance of the sample to the participants in this study, this stage was critical as the AMTMS was validated using Singapore pre-tertiary students (grade-11 and -12 students).

The review resulted in minor edits that would not impact the factorial structure of the AMTMS to three items (see Appendix A). These edits included: (1) the term “teacher” in item EMIN2 was changed to “instructors”, a term more commonly used in the university; (2) the sentence “course of my choice in university” was changed to “the subsequent courses in university” for item EMID4, as the intended participants were already enrolled in a course in university; and (3) the term “career progression” was added to item EMID1 as some of the intended participants were already working. The remaining items were considered content appropriate, based on the intended purpose of the AMTMS (i.e., to assess motivation toward mathematics based on SDT).

### 3.2. Stage 2

Following the review for content appropriateness, the AMTMS was prepared and administered via AdLeS. Ethics approval were sought from the university institution review board before participants (see Table 2) were invited via availability sampling [39] to indicate their consent preference and voluntarily complete the AMTMS at the end of their interaction with AdLeS. Participants had one month to interact with AdLeS, and would indicate participation consent by checking on a box on-screen after reading the participant information sheet online. To encourage participation, participants who completed the AMTMS were given the option to accept a cash voucher.

The age range of the participants was 20 to 67 years (*M* = 27.98, *SD* = 7.53 years). The total number of participants (*n* = 196) was considered adequate based on the recommendation by [40], which suggested the adequacy of 243 observations to estimate item and person locations based on the Rasch model.

Upon data collection, a Rasch analysis was performed using RUMM2030 (RUMM Laboratory Pty Ltd., Perth, Australia) to establish the dimensionality of the AMTMS, noting that RMT affords an evaluation of the factorial structure of an instrument via examining scores gathered through rating scales; Rasch analysis also provides information on the dimensionality and feasibility of the additivity of sub-scale scores [36,37]. As a five-factor model and multidimensionality (i.e., AMOT, EMER, EMID, EMIN and IMT factors) had previously been established via the classical factor analytic approach on the AMTMS [12], Rasch analysis was performed for each factor separately. A Rasch analysis was also performed for all items to investigate the plausibility of reporting an overall AMTMS score in addition to the five factor scores.

## 4. Results

To establish the dimensionality and validity evidence of the AMTMS in this study, fit (overall, person and item) and reliability, threshold ordering, scale targeting, item invariance (i.e., differential item functioning), dimensionality and response dependence were considered, as with most Rasch analysis reports. Essentially, items within each AMTMS factor should fit the Rasch model; this would imply that items are consistent with one another and provide invariant comparisons to ensure that factor scores characterise respondents as intended. This section reports results of each of these parameters.

### 4.1. AMTMS

Table 3 presents the fit, unidimensionality and reliability indices across the five originally purported factors of the AMTMS. Initially, the factors did not appear to fit the Rasch model well as the χ^2^ values were all statistically significant, with the exception of the AMOT and IMT factors. Nonetheless, it is noteworthy that the χ^2^ statistic is sensitive to sample size [35]. There was little evidence to suggest the presence of misfitting items, as all individual person and item fit residuals were within the range (±2.5) recommended by [41], with the exception of items EMIN2 and EMID1, which had fit residuals 3.28 and 4.26, respectively. Reliability was adequate across all factors except for the EMIN factor (PSI = 0.61; Cronbach’s alpha = 0.67), based on the recommendations of [35] (i.e., PSI > 0.7). Unidimensionality within each factor was generally supported by the results of the subtest analyses performed on the first principal component loading (PC1) derived from a principal components analysis of item residuals. Ref. [35] stated that, for non-violation of unidimensionality, subtest analyses should result in fewer than 5% of respondents showing a statistically significant difference between person locations. Other than the EMER and IMT factors that indicated 5.10% of respondents showing a statistically significant difference between person locations, results of the PC1 subtest analyses for all the other factors supported unidimensionality. As 5.10% marginally exceeded the recommended threshold, it would be tenable to suggest that the EMER and IMT factors are unidimensional. It is also evident that collectively considering the AMTMS as one scale is not tenable, given that the subtest analyses on PC1 resulted in 26.53% of respondents showing a statistically significant difference between person locations.

While the overall fit, reliability and dimensionality of the AMTMS factors were mostly consistent with the Rasch model, each of the factors displayed disordered thresholds. The presence of disordered thresholds (see Appendix B for example) suggests, amongst others, that there might be more response categories than respondents could meaningfully discriminate [37]. Subsequent analyses of factors that have items with disordered thresholds would be inappropriate and not meaningful and, hence, an adjusted scoring matrix was proposed upon an iterative process of refining the number of response categories by item, by factor. This adjusted scoring matrix is presented in Table 4.

### 4.2. AMTMS Re-Scored (AMTMS_rs_)

The AMTMS was re-scored based on the adjusted scoring matrix and a second Rasch analyses was conducted for each of the factors. Table 5 presents the fit, unidimensionality and reliability indices across the re-scored factors. Evidently, this second analysis yielded similar if not better results, along with ordered thresholds. To achieve this, however, items EMIN2 (Because I want to show to others (e.g., instructors, family, friends) that I can do mathematics) and EMID1 (Because I think that mathematics will help me better prepare for my future career/career progression) were deleted. Upon further review, removing these items was, in part, consistent with the academic motivations of adult learners (of which the sample of this study comprises), in that adult learners tend to return to school for the purposes of ensuring employability, as opposed to showing others their capabilities [42].

**Figure 1:**
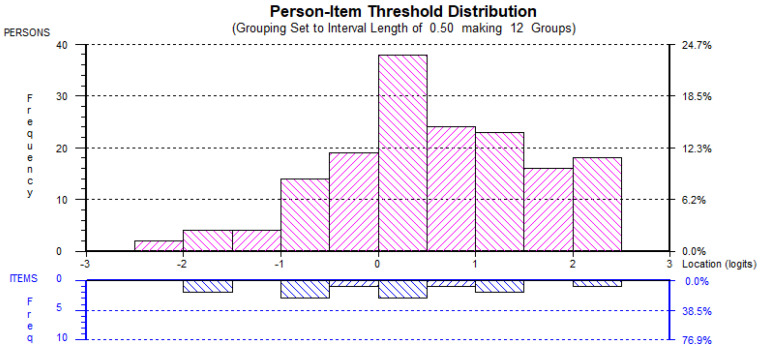
AMOT_rs_ Person-item distribution.

**Figure 2:**
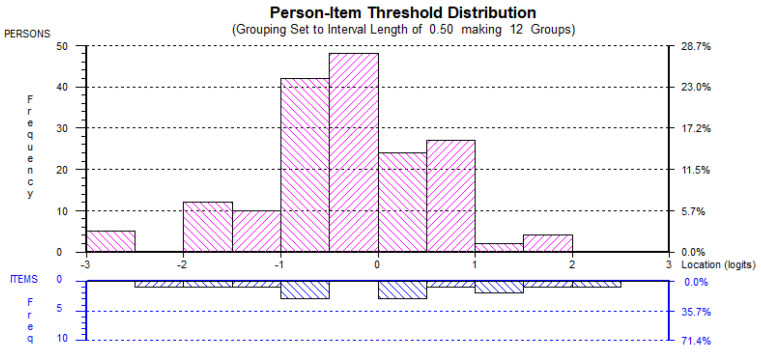
EMER_rs_ Person-item distribution.

**Figure 3:**
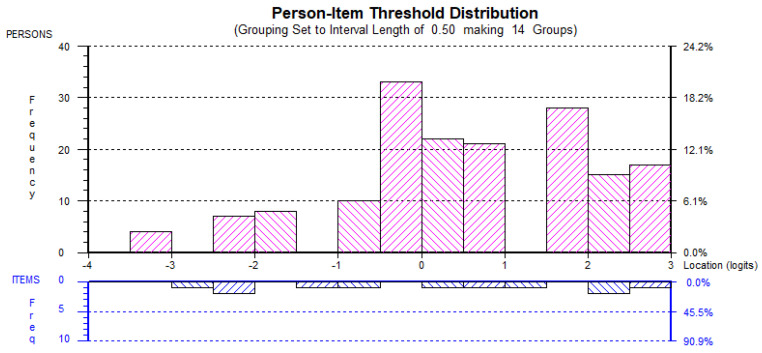
EMID_rs_ Person-item distribution.

**Figure 4:**
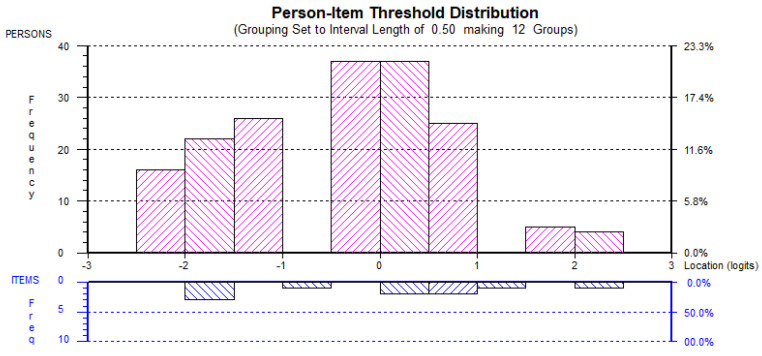
EMIN_rs_ Person-item distribution.

**Figure 5:**
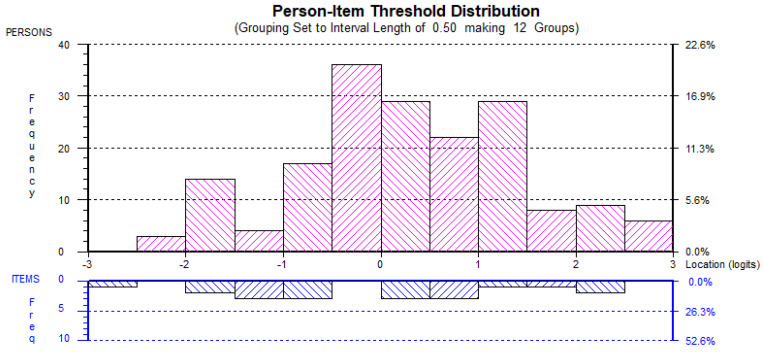
IMT_rs_ Person-item distribution.

The second Rasch analyses also found little evidence of ceiling or floor effects and, hence, it could be suggested the each of the factors were adequately targeted (see Figure 1, Figure 2, Figure 3, Figure 4 and Figure 5). All the item and Person residual means and standard deviations were close to zero and one, respectively, an indication that each of the factors were neither too easy nor difficult to endorse for the participants [41].

**Figure 6:**
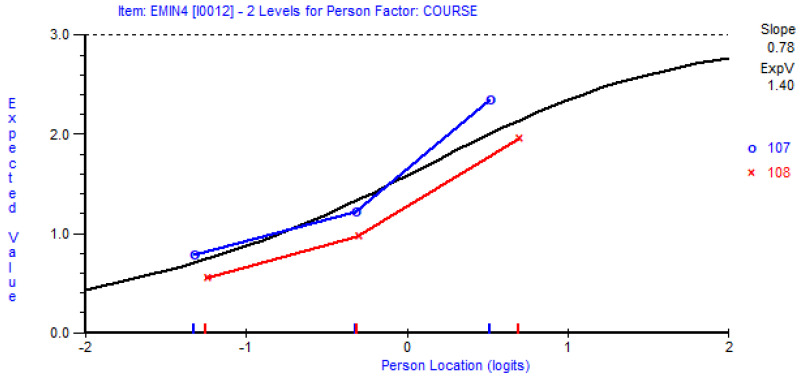
EMIN4_rs_ item characteristic curve displaying uniform DIF.

Correlation among residuals within each factor was also within the acceptable threshold of 0.3 [35], suggesting response independence between items and further supporting the unidimensionality of each factor. Item invariance was also achieved (i.e., no differential item functioning (DIF)) across both courses, with the exception of item EMIN4_rs_ (i.e., Because I want to show myself that I can do well in mathematics) (see Figure 6 for item characteristic curve), which suggested the existence of uniform DIF. Nonetheless, it is reasonable to suggest that this DIF is benign rather than adverse [43], as there is little reason to expect that students reading MTH107 would respond differently to EMIN4_rs_, compared with students reading MTH108, both of which are foundational calculus courses.

While the second Rasch analysis also found that the AMTMS_rs_ was multidimensional, an attempt at a bifactor equivalent solution by means of forming super items and subtesting each factor was made [44], with the view that reporting a singular motivation index instead of five could be more practical, particularly for policy makers. The bifactor equivalent solution performed within RUMM2030 in this instance reported a proportion of common variance of 0.82. This suggests the possibility of using a total sum-score (arising from averaging the five sub-scores of the AMTMS_rs_) as an approximation for an index representing academic motivation toward mathematics, based on common variance thresholds recommended by [45,46].

## 5. Discussion

Academic motivation remains critical and should be considered in developing adaptive learning systems [19,20,21]. With this in view, this study sought to validate a measure (i.e., the AMTMS) that would potentially provide information about student academic motivation toward mathematics as they interacted with an adaptive learning system (i.e., AdLeS). Participants were part-time adult learners reading level one mathematics courses in a tertiary educational institution. Despite an extensive Rasch analysis performed on each of the purposed factors (i.e., AMOT, EMER, EMID, EMIN and IMT factors), disordered thresholds remained. To overcome the issue of disordered thresholds, this study proposed an adjusted scoring matrix that should be used to compute item-level scores. For example, item EMER3 has a scoring strategy of 0-1-1-2-3; this would mean that response scores would correspond to “0 Does not correspond at all; 1 Corresponds a little; 1 Corresponds moderately; 2 Corresponds a lot; 3 Corresponds exactly” instead of “1 Does not correspond at all; 2 Corresponds a little; 3 Corresponds moderately; 4 Corresponds a lot; 5 Corresponds exactly”. Other indicators commonly studied in RMT (i.e., fit (overall, person and item) and reliability, scale targeting, item invariance (i.e., DIF), dimensionality and response dependence) were within recommended thresholds, though benign DIF was found for item EMIN4_rs_.

Re-scoring the AMTMS resulted in ordered thresholds but, of the 21 items, 2 had to be deleted (items EMIN2 and EMID1) to achieve model fit; this deletion was deemed tenable as the items were not as relevant for adult learners. For the purpose of reporting a singular motivation score, an approximation for an index representing academic motivation toward mathematics was sought following the Rasch analyses of each factor. Clearly, the total sum scores of all 21 AMTMS items or 19 AMTMS_rs_ items could not be taken as unidimensional. Nonetheless, a bifactor equivalent solution performed within RUMM2030 retained a proportion of common variance of 0.82 after forming super items and subtesting each factor. This suggested the possibility that, while the AMTMS or AMTMS_rs_ are not unidimensional, a bifactor solution exists and a singular motivation score could be used as an approximation of the five factors.

Based on the findings of this study, it is recommended that further bifactor equivalent analyses (with more data) be undertaken within RUMM2030, as some studies have recommended that a 0.90 proportion of common variance retained might be more conservative [46]. The potential of using the AMTMS_rs_ for a conventional class (i.e., not adaptive learning) could also be explored, and motivation factor scores could be compared, to determine if the mode (i.e., in-person lessons or adaptive learning) influences academic motivation toward mathematics.

## 6. Conclusions

This study found that all except for two AMTMS items functioned according to the RMT. Validity evidence based on internal structure found from the Rasch analyses complemented by those by [12], with the additional suggestion of using a singular motivation index to represent the five factors. Given this, the context of this study (i.e., academic motivation toward mathematics as part-time adult learners interacted with AdLeS), and the criticality of considering motivation in online learning contexts, the 19-item AMTMS_rs_ holds promise as an instrument ready for use as researchers develop adaptive learning systems. Based on the Rasch analyses, the AMTMS_rs_ would allow users to establish associations between each of the five factors (i.e., AMOT, EMER, EMIN, EMID, IMT); this information would help developers of adaptive learning systems appreciate which are potential motivation areas that students lack and, hence, be better able to help learners to achieve selected goals.

## Figures and Tables

**Table 1:** SDT: amotivation, extrinsic motivation, and intrinsic motivation.

Amotivation (AMOT): Nonself-Determined	Extrinsic Motivation * (EMOT): Least Self-Determined	Intrinsic Motivation (IMOT): Most Self-Determined
Lack of motivation	1. External regulation (EMER) (lower): reward or punishment (non-autonomous)	Perform a task due to enjoyment, interest, or satisfaction
Absence of both intrinsic and extrinsic motivations	2. Introjected regulation (EMIN): social approval or guilt (non-autonomous)	Presence of high-quality learning
	3. Identified regulation (EMID): self-endorsement of goals (autonomous)	
	4. Integrated regulation (EMIR) (higher): congruence (autonomous)	

*Note*. * SDT advocates four types of extrinsic motivation: external regulation and introjected regulation (non-autonomous); identified regulation and integrated regulation (autonomous).

**Table 2:** Participants.

Course/Semester/Year	Number of Enrolled Students *	Number of Students Who Completed the AMTMS
MTH107/2/2021	130	42
MTH107/1/2022	160	80
MTH108/2/2021	74	41
MTH108/1/2022	96	33

*Note*. * All were part-time adult learners as the university these students were enrolled in caters primarily to adult learners.

**Table 3:** Fit, unidimensionality and reliability indices of AMTMS.

Factor	Number of Items	χ^2^ Value/*p*	PSI	Cronbach’s Alpha	Unidimensionality (%)	Item Residual (*M*/*SD*)	Person Residual (*M*/*SD*)
AMOT	4	15.28/0.05	0.68	0.80	2.04	0.37/1.48	−0.49/1.19
EMER	4	23.47/0.003	0.74	0.78	5.10	0.47/1.54	−0.64/1.44
EMIN	4	53.04/0.000	0.61	0.67	3.06	0.66/1.86	−0.405/1.41
EMID	4	55.59/0.000	0.74	0.76	1.53	0.77/2.45	−0.46/1.32
IMT	5	4.48/0.92	0.82	0.86	5.10	0.25/0.48	−0.74/1.64
AMTMS	21	200.42/0.000	0.90	0.88	26.53	0.77/2.38	−0.47/2.24

*Note*. *M* = mean; *SD* = standard deviation; *p* = probability; PSI = Person separation index.

**Table 4:** Adjusted scoring matrix.

Item	Original Scoring	Adjusted Scoring
AMOT1	0-1-2-3-4	4-3-2-1-0
AMOT2	0-1-2-3-4	2-2-1-0-0
AMOT3	0-1-2-3-4	3-2-1-0-0
AMOT4	0-1-2-3-4	4-3-2-1-0
EMER1	0-1-2-3-4	0-1-2-3-4
EMER2	0-1-2-3-4	0-1-2-3-4
EMER3	0-1-2-3-4	0-1-1-2-3
EMER4	0-1-2-3-4	0-1-1-2-3
EMIN1	0-1-2-3-4	0-1-1-2-3
EMIN2	0-1-2-3-4	0-1-1-2-3
EMIN3	0-1-2-3-4	0-1-2-3-4
EMIN4	0-1-2-3-4	0-1-1-2-3
EMID1	0-1-2-3-4	0-1-1-2-3
EMID2	0-1-2-3-4	0-1-2-3-4
EMID3	0-1-2-3-4	0-1-1-2-3
EMID4	0-1-2-3-4	0-1-2-3-4
IMTA4	0-1-2-3-4	0-1-2-3-4
IMTK2	0-1-2-3-4	0-1-2-3-4
IMTK3	0-1-2-3-4	0-0-1-2-3
IMTS2	0-1-2-3-4	0-1-2-3-4
IMTS3	0-1-2-3-4	0-1-2-3-4

**Table 5:** Fit, undimensionality and reliability indices of AMTMS re-scored.

Factor	Number of items	χ^2^ Value/*p*	PSI	Cronbach’s Alpha	Unidimensionality (%)	Item Residual (*M*/*SD*)	Person Residual (*M*/*SD*)
AMOT_rs_	4	14.07/0.08	0.66	0.77	2.04	0.20/0.99	−0.43/1.14
EMER_rs_	4	20.88/0.007	0.73	0.76	5.10	0.51/1.20	−0.65/1.41
EMIN_rs_	3	12.72/0.05	0.64	0.72	2.55	0.46/0.72	−0.50/1.05
EMID_rs_	3	7.09/0.31	0.77	0.84	4.08	0.39/0.56	−0.70/1.32
IMT_rs_	5	5.52/0.85	0.82	0.97	5.10	0.29/0.63	−0.71/1.63
AMTMS_rs_	19	239.64/0.000	0.87	0.86	25.51	0.71/3.22	−0.56/2.40

*Note. M* = mean; *SD* = standard deviation; *p* = probability; PSI = Person separation index; rs = rescored based on adjusted scoring matrix.

**Figure A1:**
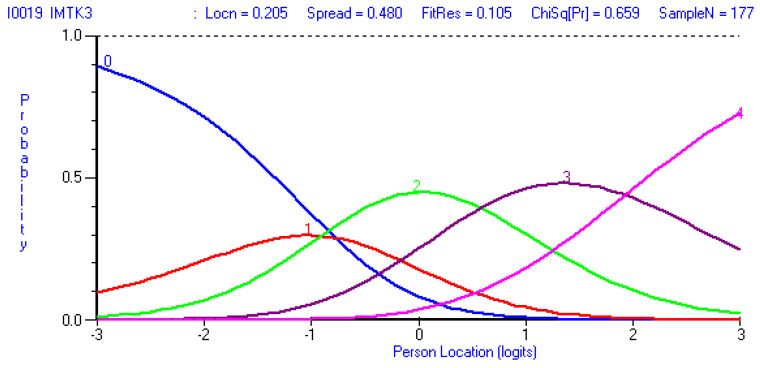
In this figure, evidence of a disordered threshold is presented in I0019 before scoring is adjusted. Category 1 (“corresponds a little”) never has maximal probability and would unlikely be selected by any respondent, regardless of person endorsability (i.e., logit). This suggests that the item categories are not functioning as intended.

**Figure A2:**
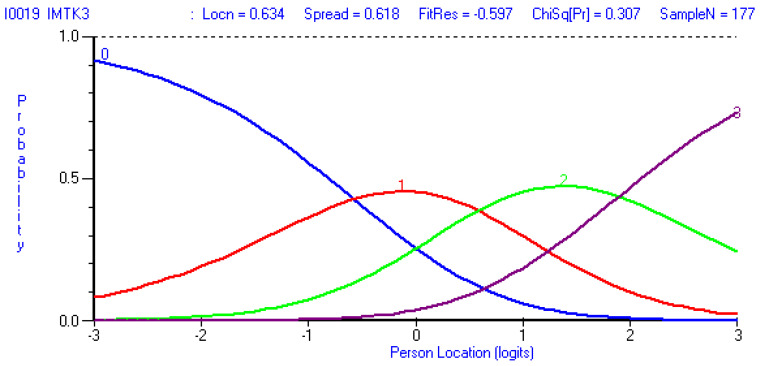
In this figure (after applying the adjusted scoring matrix), it is evident that a person higher on the AMTMS scale displays a higher probability of selecting a higher category. To illustrate, a person of 0 and 1 logit on the AMTMS scale would have a higher probability of selecting category 1 (“corresponds a little”) and category 2 (“corresponds moderately”) to this item, respectively.

## Data Availability

Data available on request due to privacy and ethical restrictions.

## Appendix A. Academic Motivation toward Mathematics Scale (5 Point Likert Scale: 1, Does Not Correspond at All; 2, Corresponds a Little; 3, Corresponds Moderately; 4, Corresponds a Lot; 5, Corresponds Exactly)

Why do you want to spend time studying Mathematics?

AMOT1—Honestly, I don’t know; I feel that it is a waste of time studying mathematics.

EMIN2—Because I want to show to others (e.g., instructors, family, friends) that I can do mathematics.

EMIN4—Because I want to show myself that I can do well in mathematics.

AMOT4—I am not sure; I don’t see how mathematics is of value to me.

EMER1—Because without a good grade in mathematics, I will not be able to find a high-paying job later on.

EMID3—Because I believe that mathematics will improve my work competence.

IMTK2—For the pleasure I experience when I discover new things in mathematics that I have never learnt before.

EMER2—In order to obtain a more prestigious job later on.

EMIN3—To show myself that I am an intelligent person.

EMER4—In order to have a better salary later on.

IMTS3—For the pleasure that I experience when I feel completely absorbed by what mathematicians have come up with.

EMID4—Because what I learn in mathematics now will be useful for the subsequent courses in university.

IMTA4—Because I want to feel the personal satisfaction of understanding mathematics.

EMID2—Because studying mathematics will be useful for me in the future.

IMTK3—For the pleasure that I experience in broadening my knowledge about mathematics.

EMER3—Because I want to have “the good life” later on.

AMOT3—I don’t know; I can’t understand what I am doing in mathematics.

EMID1—Because I think that mathematics will help me better prepare for my future career/career progression.

AMOT2—I can’t see why I study mathematics and frankly, I couldn’t care less.

EMIN1—Because of the fact that when I do well in mathematics, I feel important.

IMTS2—For the pleasure that I experience when I learn how things in life work, because of mathematics.

## References

[1] Yarnall L., Means B., Wetzel T. (2016). Lessons Learned from Early Implementations of Adaptive Courseware.

[2] Booth C., Cheluvappa R., Bellinson Z., Maguire D., Zimitat C., Abraham J., Eri R. (2016). Empirical evaluation of a virtual laboratory approach to teach lactate dehydrogenase enzyme kinetics. Ann. Med. Surg..

[3] Förster M., Weiser C., Maur A. (2018). How feedback provided by voluntary electronic quizzes affects learning outcomes of university students in large classes. Comput. Educ..

[4] Liu M., McKelroy E., Corliss S.B., Carrigan J. (2017). Investigating the effect of an adaptive learning intervention on students’ learning. Educ. Technol. Res. Dev..

[5] Santos J.L., Govaerts S., Verbert K., Duval E. Goal-oriented visualizations of activity tracking: A case study with engineering students. Proceedings of the 2nd International Conference on Learning Analytics and Knowledge.

[6] Demski J. (2012). This time it’s personal. Technol. Horiz. Educ..

[7] Johnson L., Adams Becker S., Cummins M., Estrada V., Freeman A., Hall C. (2016). NMC Horizon Report. https://www.learntechlib.org/p/171478/.

[8] Garrick B., Pendergast D., Geelan D. (2017). Austin, Texas: The New Media Consortium. Introduction to the philosophical arguments underpinning personalised education. Theorising Personalised Education.

[9] Wolper J. (2016). Student-driven personalized learning is trending in higher education. Talent Dev..

[10] Felt L.J., Robb M.B. (2016). Technology Addiction: Concern, Controversy, and Finding Balance.

[11] Male T., Burden K. (2014). Access denied? Twenty-first-century technology in schools. Technol. Pedagog. Educ..

[12] Lim S.Y., Chapman E. (2015). Adapting the academic motivation scale for use in pre-tertiary mathematics classrooms. Math. Educ. Res. J..

[13] Ryan R.M., Deci E.L. (2017). Self-Determination Theory: Basic Psychological Needs in Motivation Development and Wellness.

[14] Pintrich P. (2003). A motivational science perspective on the role of student motivation in learning and teaching contexts. J. Educ. Psychol..

[15] Dweck C.S. (2017). From needs to goals and representations: Foundations for a unified theory of motivation, personality, and development. Psychol. Rev..

[16] Ryan R.M., Deci E.L. (2000). Self determination theory and the facilitation of intrinsic motivation, social development, and well-being. Am. Psychol..

[17] Schunk D.H., Usher E.L., Ryan R.M. (2012). Social cognitive theory and motivation. The Oxford Handbook of Human Motivation.

[18] Pantziara M., Philippou G.N. (2015). Students’ motivation in the mathematics classroom revealing causes and consequences. Int. J. Sci. Math. Educ..

[19] Joo Y.J., Oh E., Kim S.M. (2015). Motivation, instructional design, flow, and academic achievement at a Korean online university: A structural equation modeling study. J. Comput. High. Educ..

[20] Moos D.C., Bonde C. (2016). Flipping the classroom: Embedding self-regulated learning prompts in videos. Technol. Knowl. Learn..

[21] Kim C.M., Park S.W., Cozart J. (2014). Affective and motivational factors of learning in online mathematics courses. Br. J. Educ. Technol..

[22] Artino A.R. (2008). Motivational beliefs and perceptions of instructional quality: Predicting satisfaction with online training. J. Comput. Assist. Learn..

[23] Keller J.M. (2008). First principles of motivation to learn and e^3^-learning. Distance Educ..

[24] Ryan R.M., Deci E.L. (2020). Intrinsic and extrinsic motivation from a self-determination theory perspective: Definitions, theory, practices, and future directions. Contemp. Educ. Psychol..

[25] Deci E.L., Ryan R.M. (2008). Self-determination theory: A macrotheory of human motivation, development, and health. Can. Psychol..

[26] Baumeister R.F., Leary M.R. (1995). The need to belong: Desire for interpersonal attachments as a fundamental human motivation. Psychol. Bull..

[27] DeCharms R. (1968). Personal Causation: The Internal Affective Determinants of Behavior.

[28] Vallerand R.J., Zanna M.P. (1997). Toward a hierarchical model of intrinsic and extrinsic motivation. Advances in Experimental Social Psychology.

[29] Butler K.L. (2016). Motivation for Mathematics: The Development and Initial Validation of an Abbreviated Instrument. Ph.D. Thesis.

[30] Vallerand R.J., Pelletier L.G., Blais M.R., Brire N.M., Sencal C., Vallires E.F. (1992). The academic motivation scale: A measure of intrinsic, extrinsic, and amotivation in education. Educ. Psychol. Meas..

[31] Can G. (2015). Turkish version of the Academic Motivation Scale. Psychol. Rep. Employ. Psychol. Mark..

[32] Staribratov I., Babakova L. (2019). Development and validation of a Math-specific version of the Academic Motivation Scale (AMS-Mathematics) among first-year university students in Bulgaria. TEM J..

[33] Aydin S., Yerdelen S., Yalmanci G.S., Göksu V. (2014). Academic Motivation Scale for learning Biology: A scale development study. Educ. Sci..

[34] Hussein J.H., Alsawaie O., Alsartawi A., Alghazo I., Tibi S. (2011). Developing Mathematics Motivation Scale for the United Arab Emirates. J. Educ. Psychol. Stud..

[35] Andrich D., Marais I. (2019). A Course in Rasch Measurement Theory: Measuring in the Educational, Social and Health Sciences.

[36] Lim L. (2022). Validation of the Moral Reasoning Questionnaire against Rasch measurement theory. J. Pac. Rim Psychol..

[37] Zhao J., Lim L., Chapman E., Houghton S. (2022). Validation of the Mental Health Changes Indicators Scale against Rasch measurement theory. Soc. Behav. Personal. Int. J..

[38] American Educational Research Association, American Psychological Association, National Council on Measurement in Education (2014). Standards for Educational and Psychological Testing.

[39] Frey B. (2018). The SAGE Encyclopedia of Educational Research, Measurement, and Evaluation.

[40] Linacre J.M. (1994). Sample size and item calibration stability. Rasch Meas. Trans..

[41] Tennant A., Conaghan P.G. (2007). The Rasch measurement model in rheumatology: What is it and why use it? When should it be applied, and what should one look for in a Rasch paper?. Arthritis Care Res..

[42] Ho Y.Y., Lim L. (2021). Targeting student learning needs: The development and preliminary validation of the Learning Needs Questionnaire for a diverse university student population. High. Educ. Res. Dev..

[43] Breslau J., Javaras K.N., Blacker D., Murphy J.M., Normand S.T. (2008). Differential item functioning between ethnic groups in the epidemiological assessment of depression. J. Nerv. Ment. Dis..

[44] An M., Yu X. (2021). A Rasch analysis of emerging adults’ health motivation questionnaire in higher education context. PLoS ONE.

[45] Andrich D. (2016). Components of variance of scales with a bifactor subscale structure from two calculations of α. Educ. Meas. Issues Pract..

[46] Rodriguez A., Reise S.P., Haviland M.G. (2016). Evaluating bifactor models: Calculating and interpreting statistical indices. Psychol. Methods.

